# Supplementary figures and images for: A scalable and tunable platform for functional interrogation of peptide hormones in fish
Source: eLife. 2023 Oct 24;12:e85960. doi: 10.7554/eLife.85960 (PMC10597582; doi:10.7554/eLife.85960)

## Figure 1- Source Data

### A. Live images of male and female WT and *gh1*<sup>Δ4/Δ4</sup>

♂

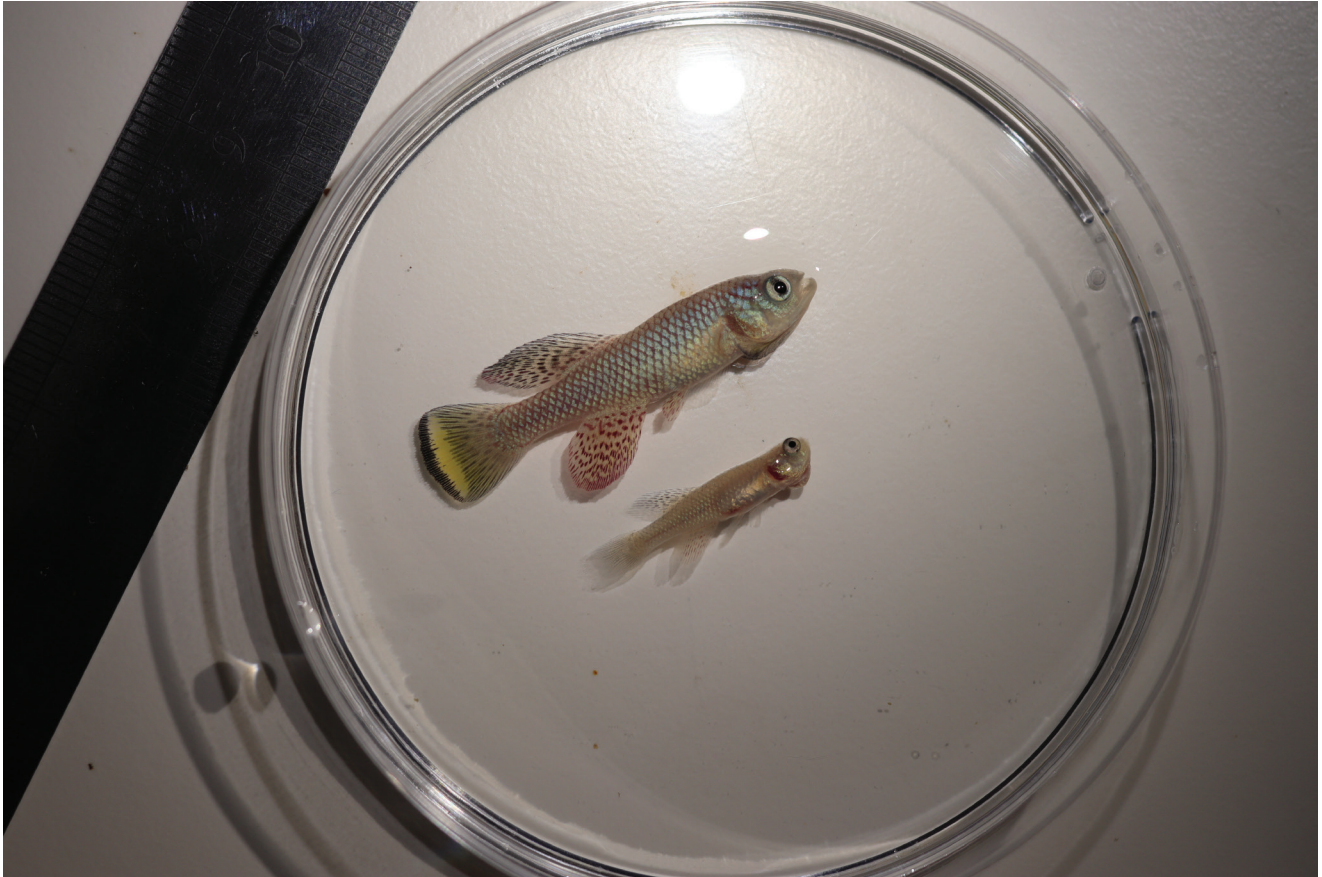

♀

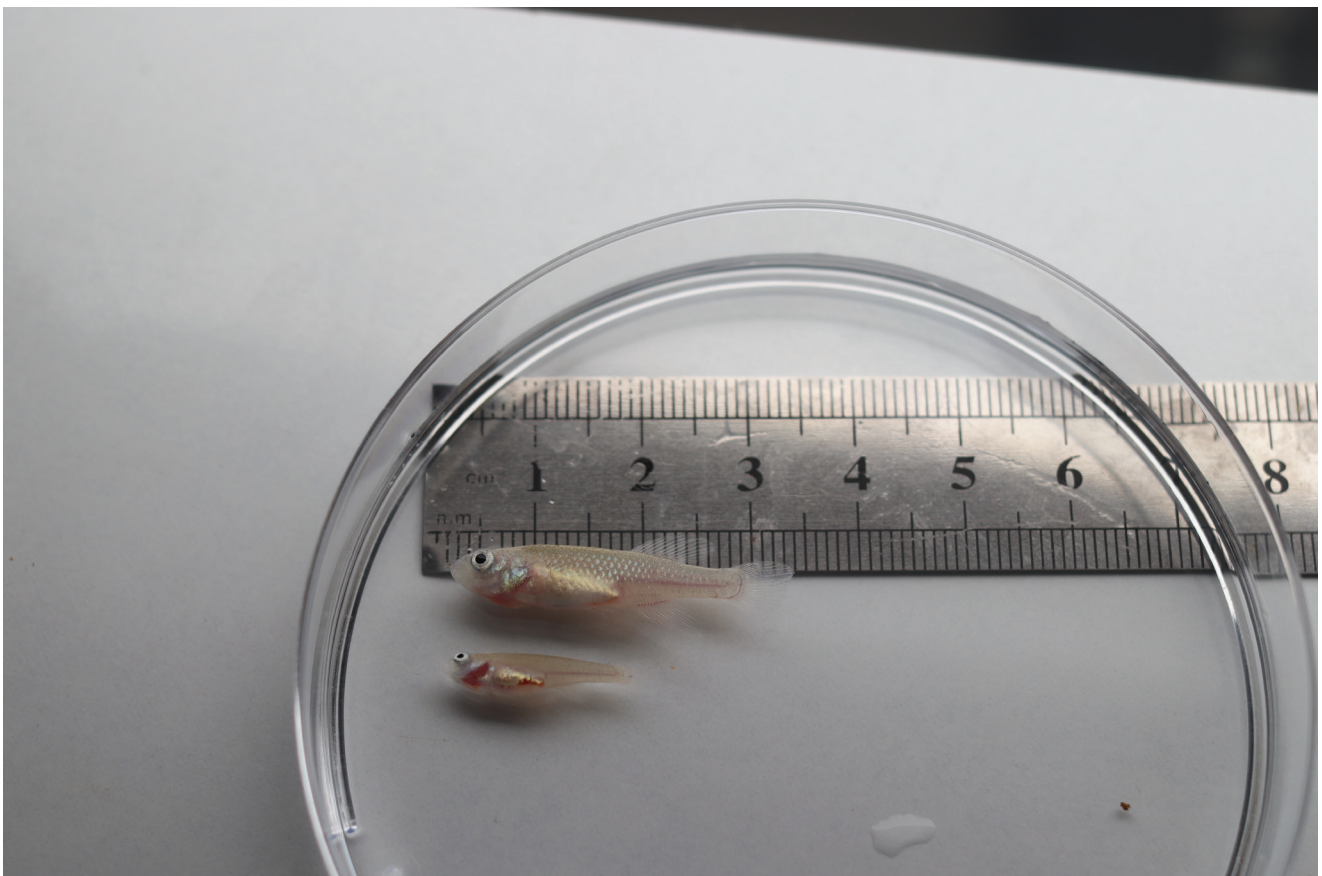

Supplement: Figure 1—source data 1. — (A) Corresponding to Figure 1B. [file elife-85960-fig1-data1.pdf]

Figure 2 - Source Data 1

A. H&E image of WT male and female gonads

♂

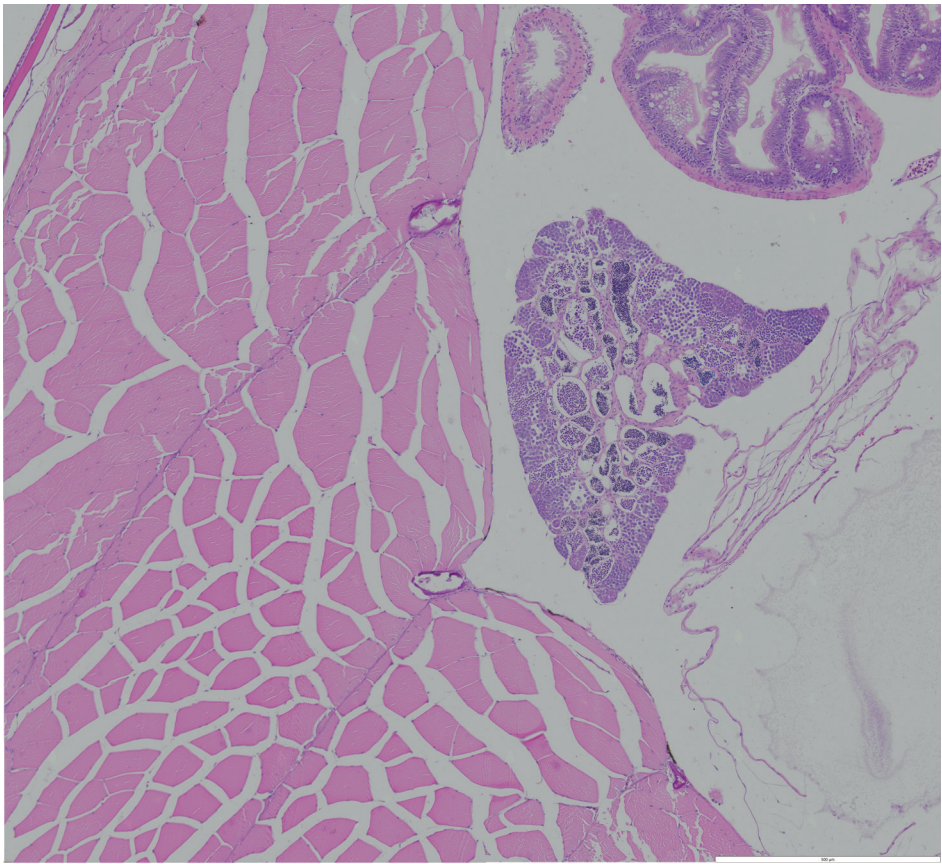

♀

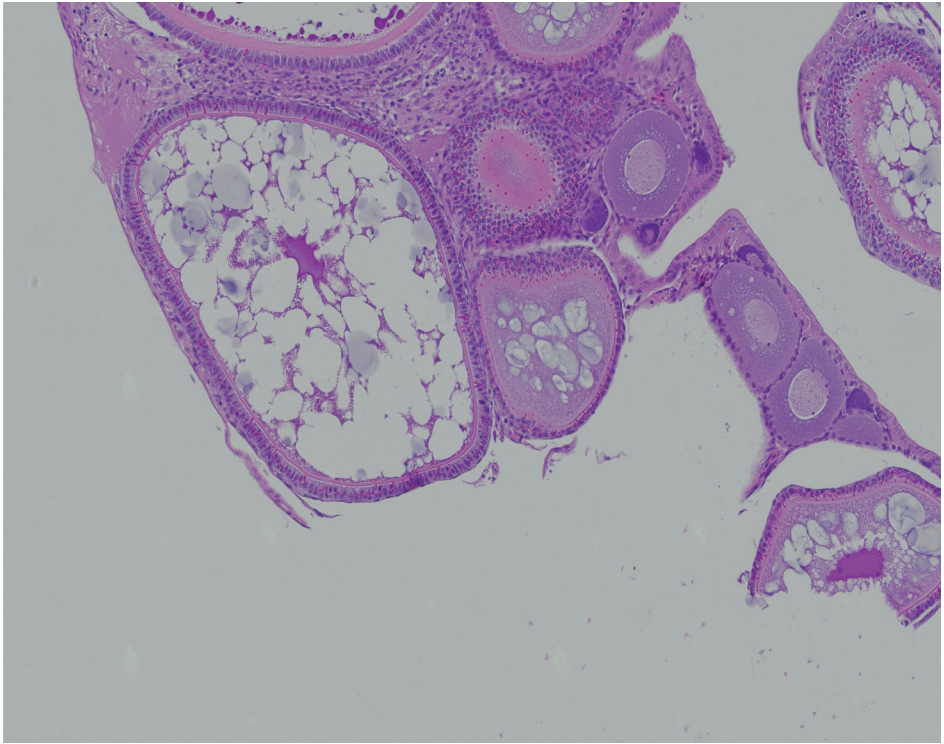

Supplement: Figure 2—source data 1. — (A) Corresponding to WT panels in Figure 2D. [file elife-85960-fig2-data1.pdf]

## Figure 2 - Source Data 2

### A. H&E image of *gh1*<sup>Δ4/Δ4</sup> male and female body cavity

♂

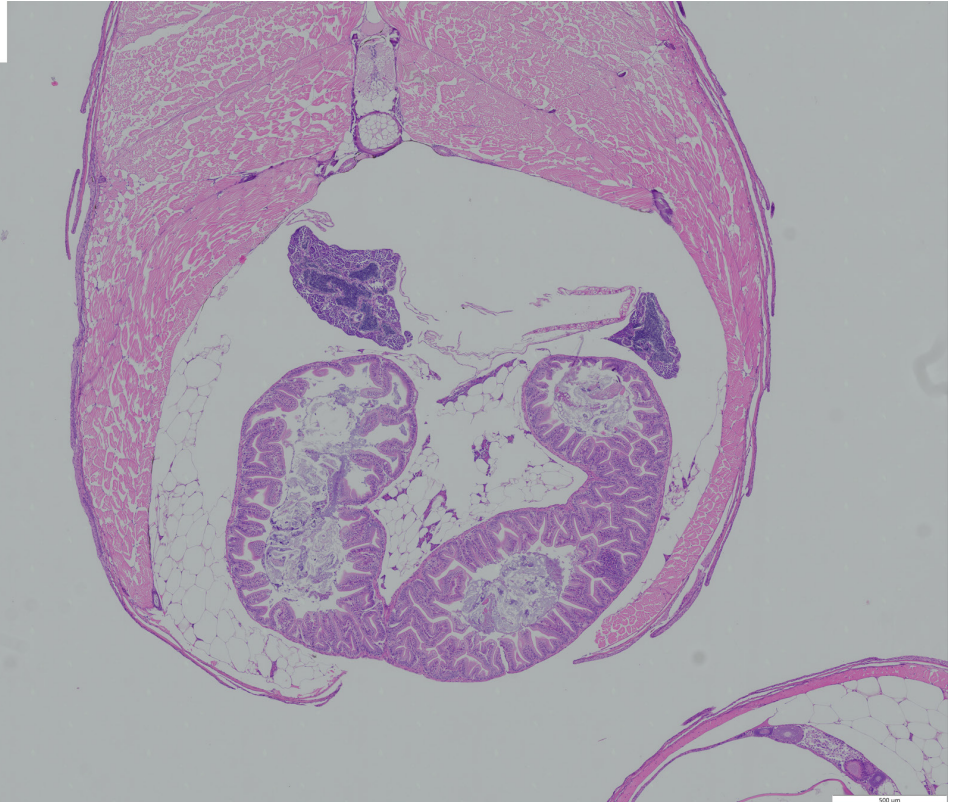

♀

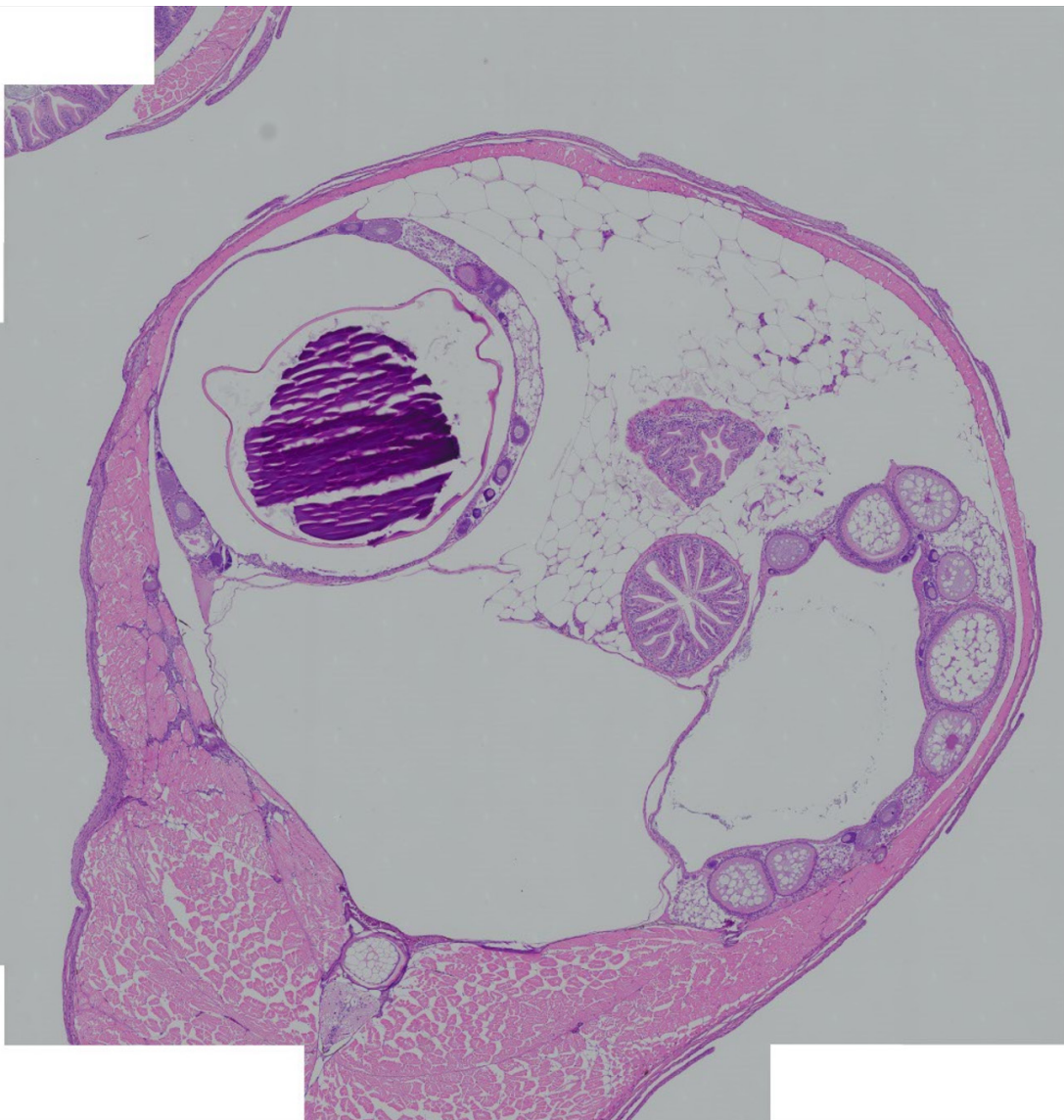

Supplement: Figure 2—source data 2. — (A) Corresponding to gh1Δ4/Δ4 panels in Figure 2D. [file elife-85960-fig2-data2.pdf]
