# Supplementary material for: A scalable and tunable platform for functional interrogation of peptide hormones in fish
Source: eLife. 2023 Oct 24;12:e85960. doi: 10.7554/eLife.85960 (PMC10597582; doi:10.7554/eLife.85960)

Figure 2 - Source Data 3

A. H&E images of the body cavity of *gh1*$^{\Delta 4/\Delta 4}$ and WT females

gh1$^{\Delta 4/\Delta 4}$ WT

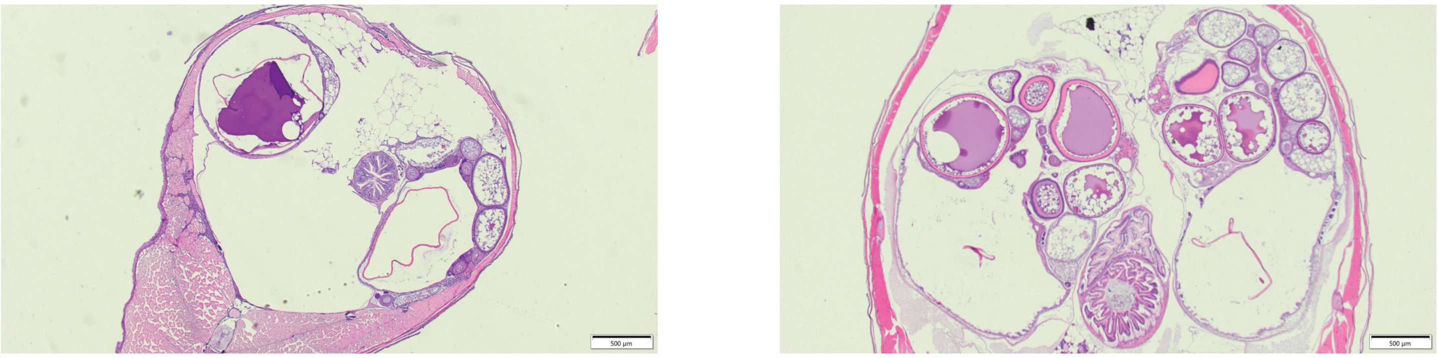

B. Live images of eggs from *gh1*$^{\Delta 4/\Delta 4}$, *gh1*$^{\Delta 4/+}$ and WT females

gh1$^{\Delta 4/\Delta 4}$ *gh1*$^{\Delta 4/+}$ WT

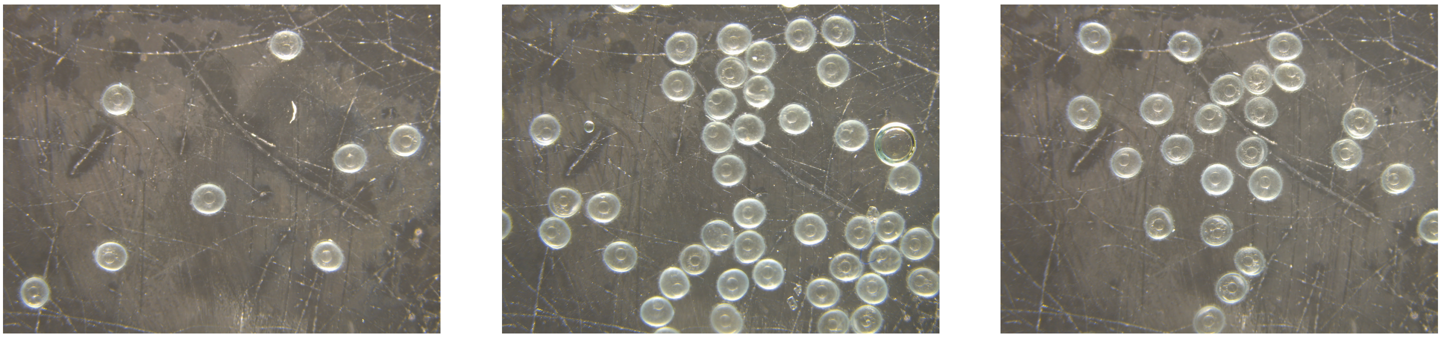

Supplement: Figure 2—figure supplement 1—source data 1. — (A) Corresponding to Figure 2—figure supplement 1A. (B) Corresponding to Figure 2—figure supplement 1B. [file elife-85960-fig2-figsupp1-data1.pdf]
