# Supplementary material for: A scalable and tunable platform for functional interrogation of peptide hormones in fish
Source: eLife. 2023 Oct 24;12:e85960. doi: 10.7554/eLife.85960 (PMC10597582; doi:10.7554/eLife.85960)

Figure 3 - Source Data 1

A. Fish injected with a *CMV:GFP* plasmid

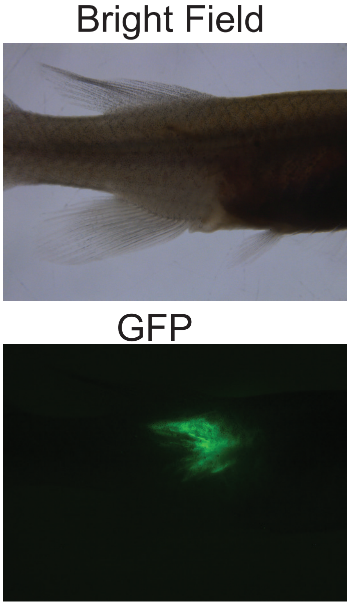

B. Fluorescent images of electroporated and non-electroporated injected fish

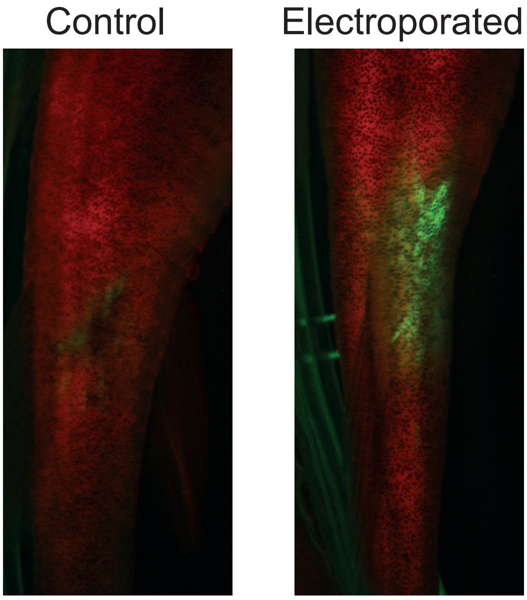

C. Fluorescent images of immunostaining for GH in muscle fibers of WT fish injected with a *CMV:gh1-T2A-GFP* plasmid with anti-GH antibody

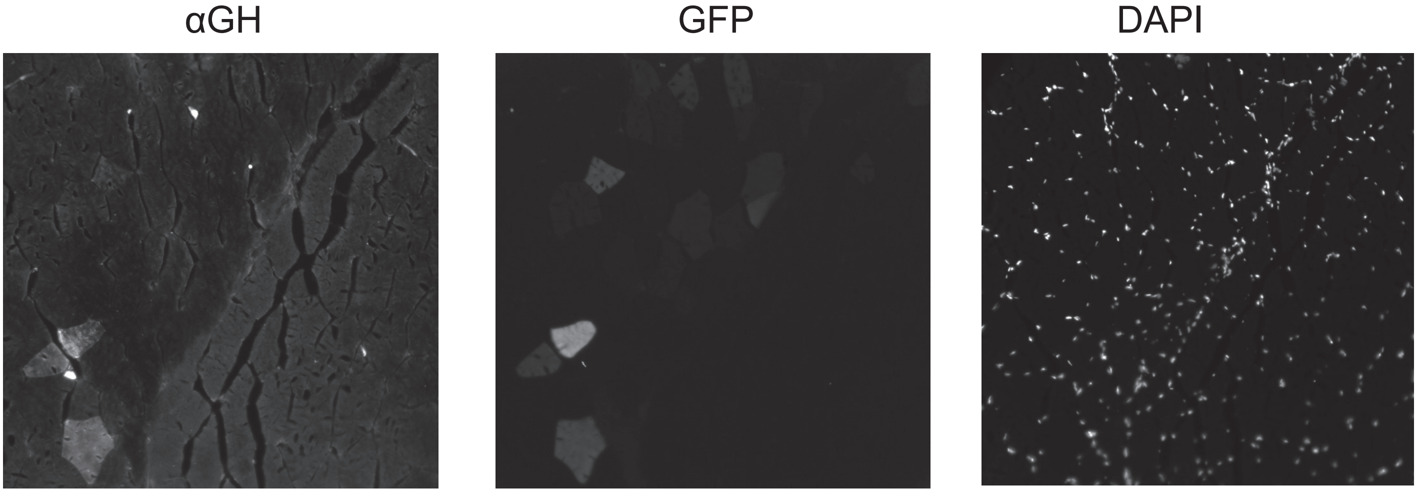

D. Fluorescent images of immunostaining for GH in muscle fibers of WT fish injected with a *CMV:gh1-T2A-GFP* plasmid with no primary antibody

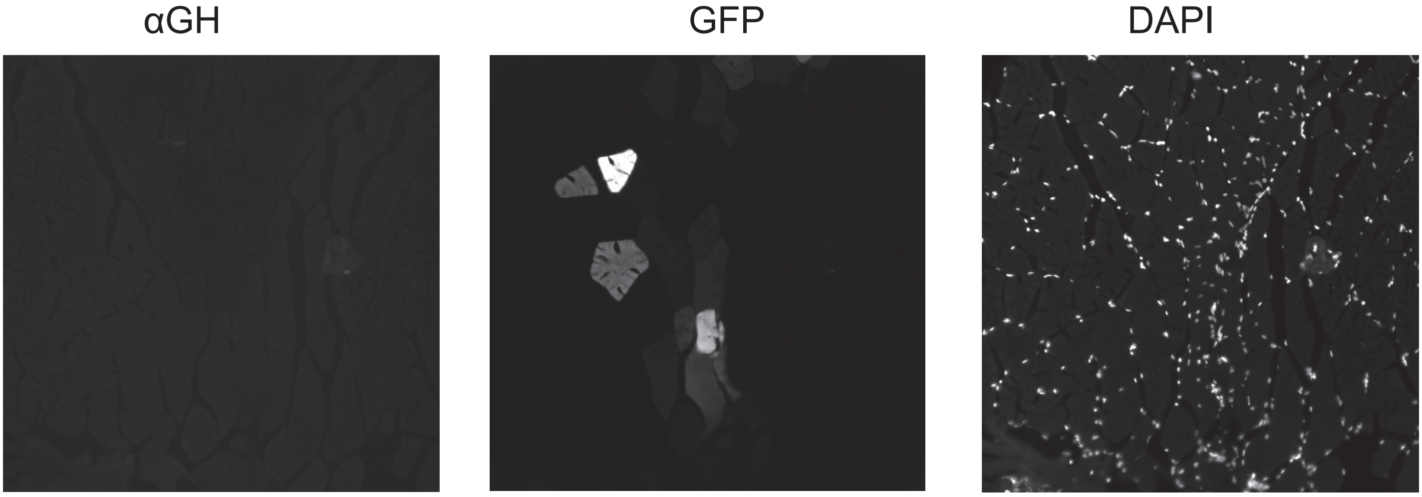

Supplement: Figure 3—source data 1. — (A) Corresponding to Figure 3A. (B) Corresponding to Figure 3B. (C) Corresponding to αGH panels in Figure 3C. (D) Corresponding to no-primary panels in Figure 3C. [file elife-85960-fig3-data1.pdf]
