# Supplementary material for: A scalable and tunable platform for functional interrogation of peptide hormones in fish
Source: eLife. 2023 Oct 24;12:e85960. doi: 10.7554/eLife.85960 (PMC10597582; doi:10.7554/eLife.85960)

Figure 3 - Source Data 2

A. Live images of male and female $gh1^{\Delta4/\Delta4}$, rescued $gh1^{\Delta4/\Delta4}$ and WT fish

$gh1^{\Delta4/\Delta4}$ | rescued $gh1^{\Delta4/\Delta4}$ | WT

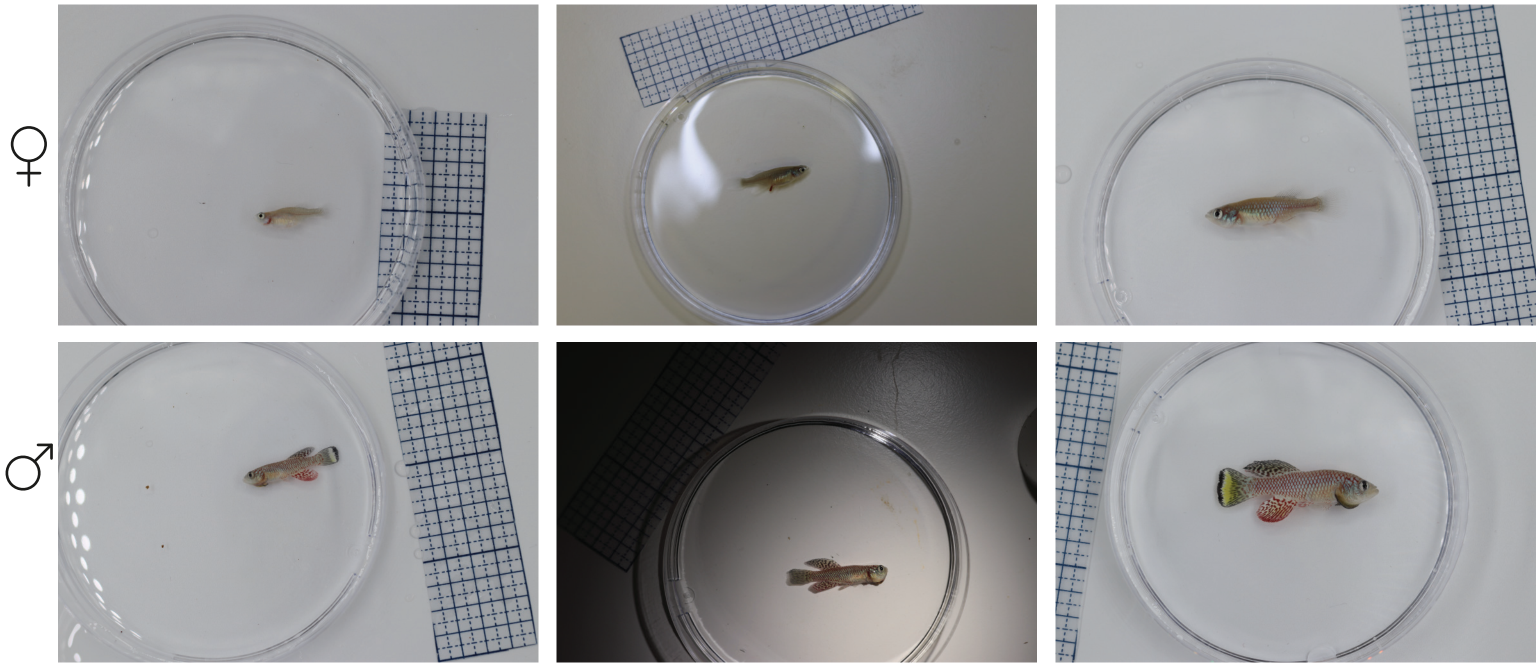

B. Live images of ovaries of $gh1^{\Delta4/\Delta4}$, rescued $gh1^{\Delta4/\Delta4}$ and WT fish

$gh1^{\Delta4/\Delta4}$ | rescued $gh1^{\Delta4/\Delta4}$ | WT

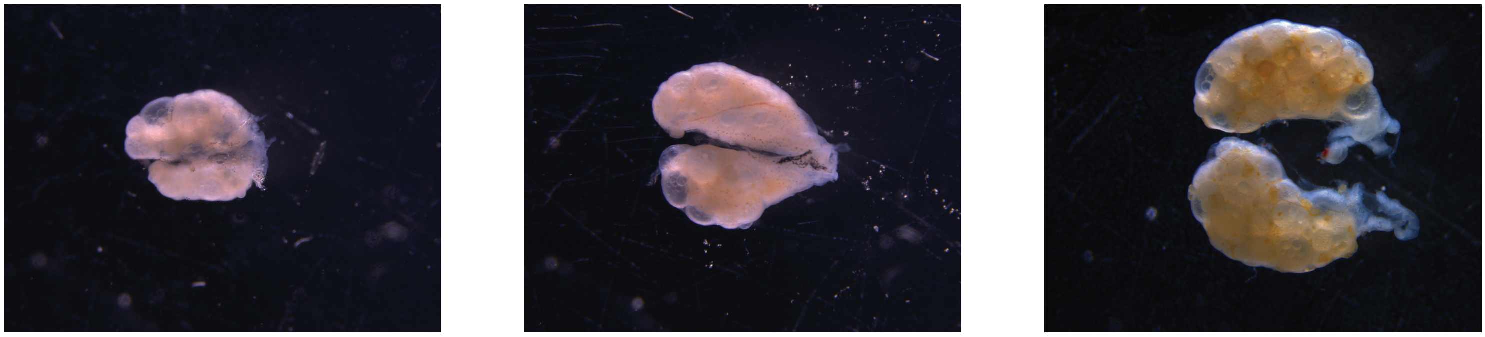

Supplement: Figure 3—source data 2. — (A) Corresponding to Figure 3D. (B) Corresponding to Figure 3F. [file elife-85960-fig3-data2.pdf]
