# Supplementary material for: A scalable and tunable platform for functional interrogation of peptide hormones in fish
Source: eLife. 2023 Oct 24;12:e85960. doi: 10.7554/eLife.85960 (PMC10597582; doi:10.7554/eLife.85960)

Figure 3 - Source Data 3

A. Fluorescent images of immunostaining for GH in muscle fibers of *gh1*$^{\Delta4/\Delta4}$ fish injected with the indicated plasmids

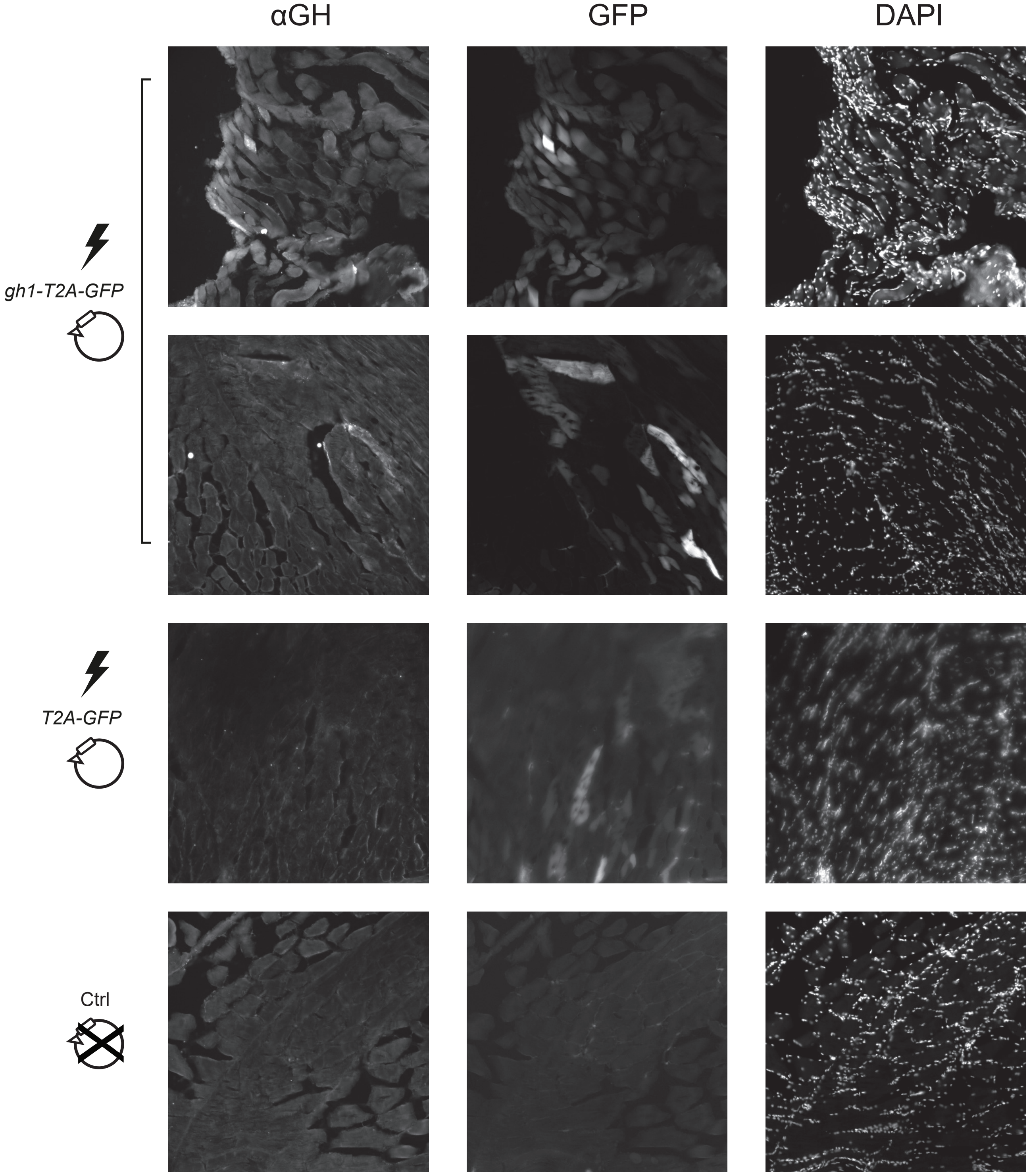

Supplement: Figure 3—figure supplement 1—source data 1. — (A) Corresponding to Figure 3—figure supplement 1A. [file elife-85960-fig3-figsupp1-data1.pdf]
