# Supplementary material for: A scalable and tunable platform for functional interrogation of peptide hormones in fish
Source: eLife. 2023 Oct 24;12:e85960. doi: 10.7554/eLife.85960 (PMC10597582; doi:10.7554/eLife.85960)

Figure 4 - Source Data 1

A. Live images of WT and *tshb*$^{\Delta 10/\Delta 10}$ male fish

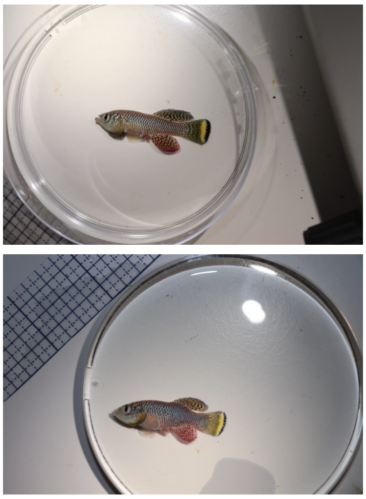

B. H&E images of WT and *tshb*$^{\Delta 10/\Delta 10}$ ovary

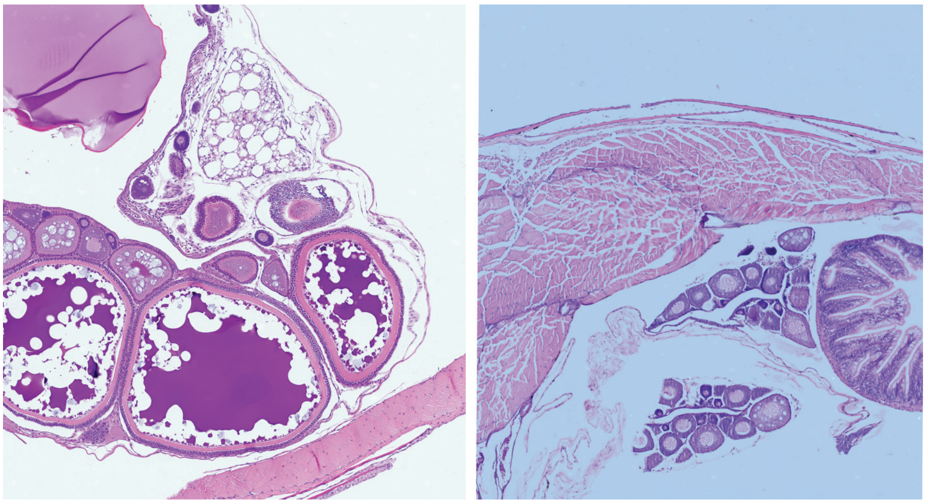

C. H&E images of WT and *fshb*$^{in1/in1}$ ovary

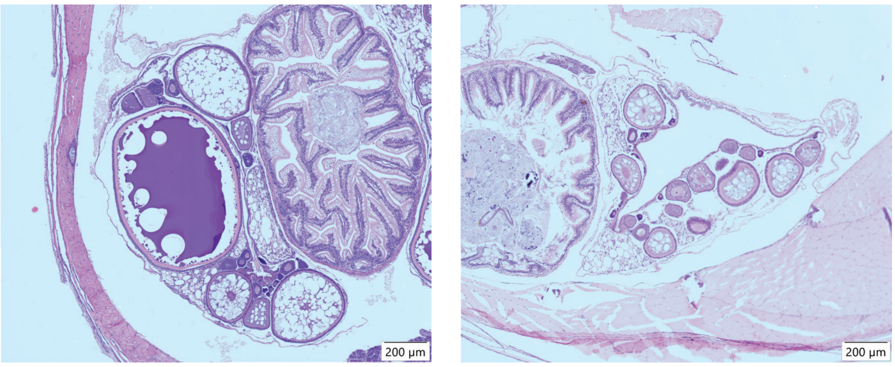

D. H&E image of rescued *fshb*$^{in1/in1}$ ovary

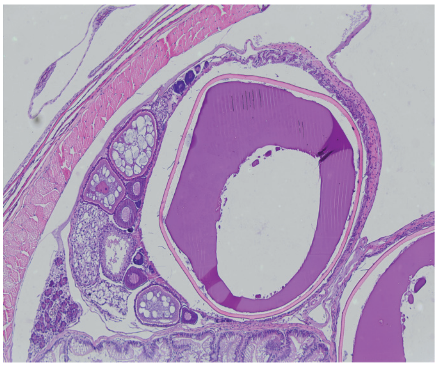

E. live and fluorescent images of ovaries of fish injected with an *fshb-GFP* fusion plasmid

Bright field

GFP

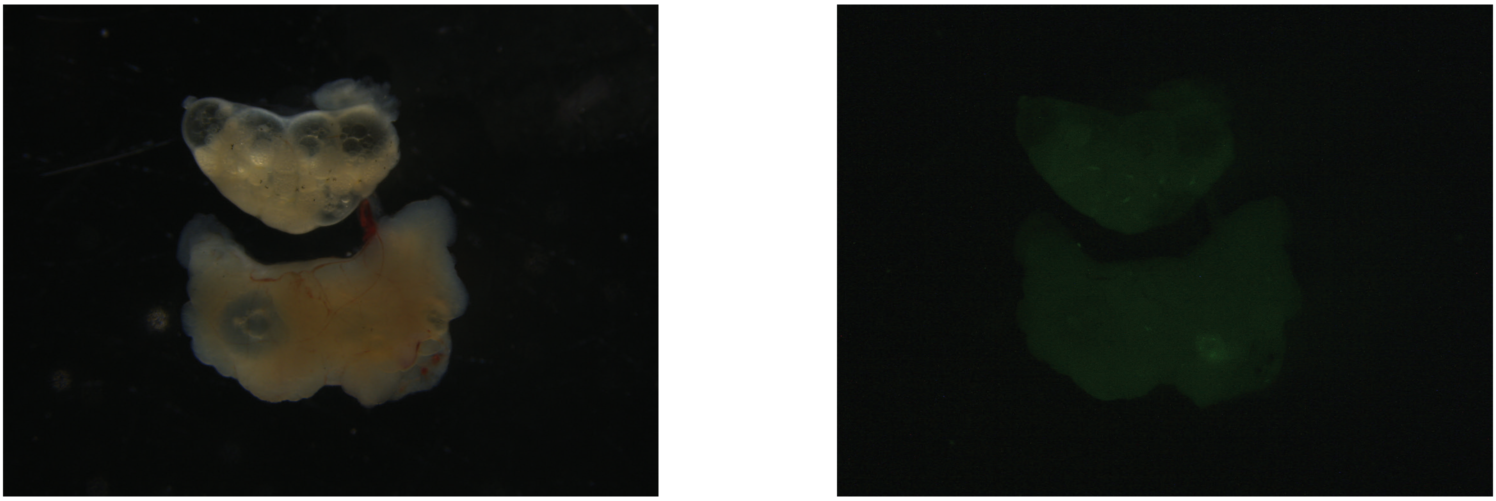

Supplement: Figure 4—source data 1. — (A) Corresponding to live image panels in Figure 4B. (B) Corresponding to H&E staining panels in Figure 4B. (C) Corresponding to Figure 4D. (D) Corresponding to Figure 4E. (E) Corresponding to Figure 4—figure supplement 1C. [file elife-85960-fig4-data1.pdf]
