# Supplementary material for: A scalable and tunable platform for functional interrogation of peptide hormones in fish
Source: eLife. 2023 Oct 24;12:e85960. doi: 10.7554/eLife.85960 (PMC10597582; doi:10.7554/eLife.85960)

Figure 5 - Source Data 1

A. Fluorescent images of fish injected with different amounts of a *CMV: gh1-T2A-GFP* plasmid

3ug

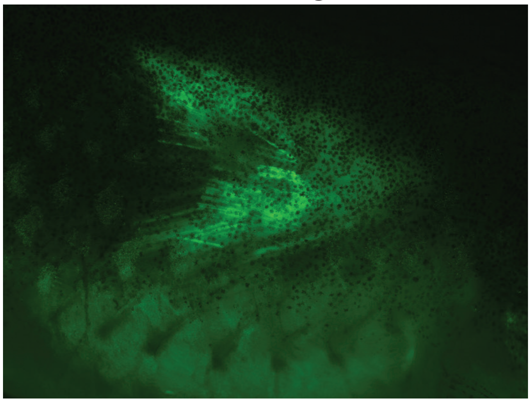

600 ng

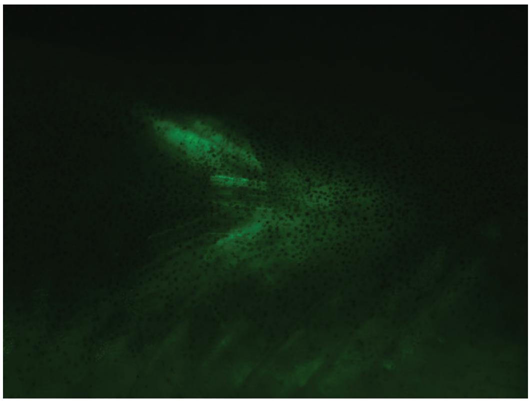

120 ng

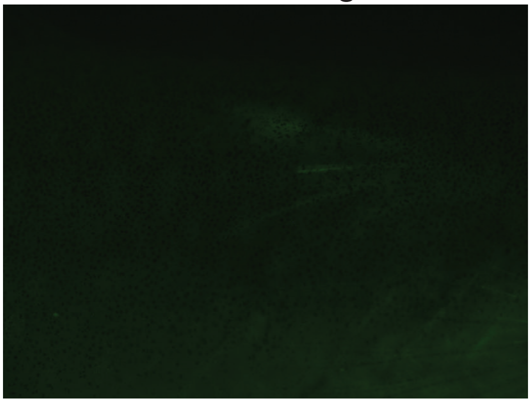

24 ng

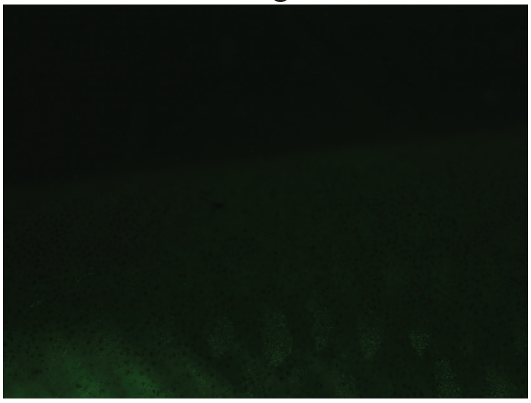

B. Fluorescent images of fish injected with *gh1-GFP and fshb-dTomato*

GFP

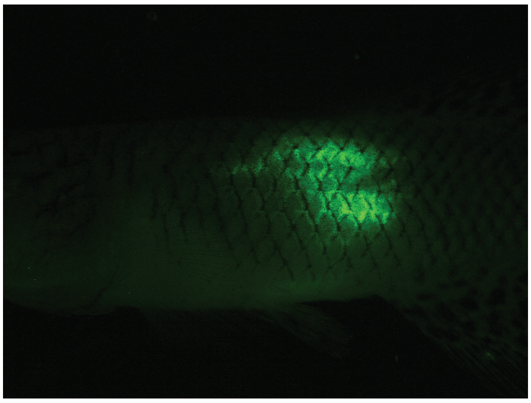

dTomato

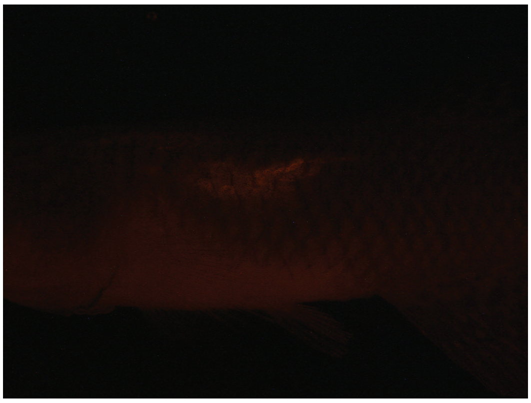

C. Fluorescent images of TetOn:fshb-T2A-GFP electroporated fish with and withot Dox treatment

\- Dox

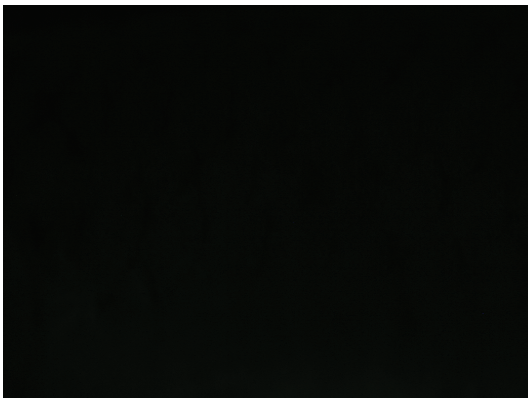

+Dox

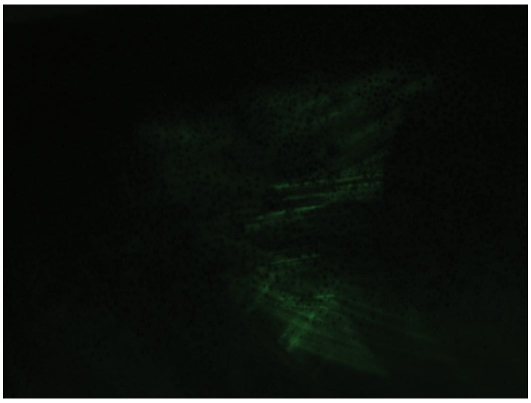

Supplement: Figure 5—source data 1. — (A) Corresponding to top panels in Figure 5B. (B) Corresponding to bottom panel in Figure 5B. (C) Corresponding to Figure 5E. [file elife-85960-fig5-data1.pdf]
